# Lars R Holsti (1926–2023), Big name in Finnish and Nordic oncology

**DOI:** 10.2340/1651-226X.2024.40168

**Published:** 2024-04-29

**Authors:** Matti Mäntylä, Inkeri Elomaa, Johanna Mattson, Pia Lindroos, Dick Killander, Bo Littbrand, Jens Overgaard, Mikko Tenhunen

**Affiliations:** a Helsinki University Hospital, Comprehensive Cancer Centre, Finland; b Helsinki, Finland; c Lund University, Sweden; d Umeå University, Sweden; e Aarhus University, Denmark

**Figure:**
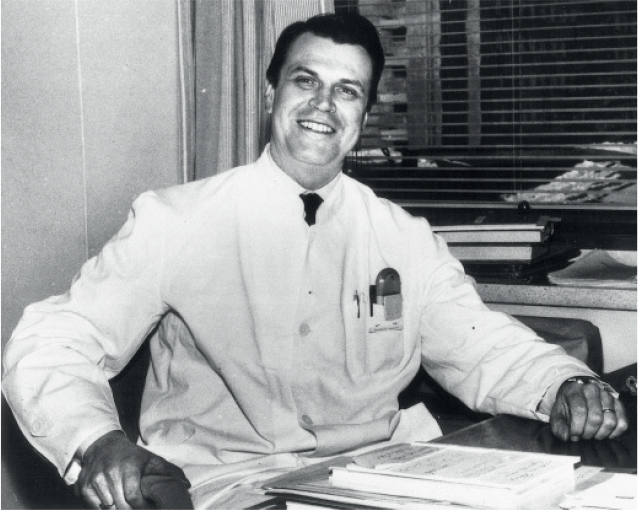


Lars Holsti, professor emeritus of oncology, died in Helsinki on December 20, 2023. He was born in Haapajärvi on March 05, 1926. Holsti’s childhood in Haapajärvi was cut short by the sudden death of his father. The family had to move to Helsinki. He entered the medical faculty in 1948. Specialization in cancer treatment was a matter of course for him.

In the early years of his career, he married Ulla (née Ramström) and they had two children. In 1968, he was appointed Professor of Radiation therapy and Chief physician of the Department of Radiation Oncology of the Helsinki University Hospital.

Holsti was an internationally distinguished specialist in cancer with high expertise in radiation biology. When he became a professor, there was no formal speciality in radiation therapy and oncology in Finland. Radiation therapy was administered by radiologists. Cancer treatment did not develop, and the number of radiotherapy equipment and medical doctors per capita was less than in the other Nordic countries. The situation improved when medical oncology and radiotherapy methods developed [1].

Professor Erik Poppe from Oslo invited Nordic professors for a meeting to standardize training of oncologists in 1971, and their report was finalized in the following year. After this the standardization had to be carried out in each country. Holsti was a key player in developing training of the oncologists in Finland [2], and together with Jerzy Einhorn from Stockholm, they pushed the specialization to cover both radiation and medical oncology, contrary to several other western countries. As the vice president of the Finnish Medical Board’s Cancer program (1988–1993), he had the big task of building specialist training and qualification requirements. This was followed by harmonization with the other Nordic countries and later with the EU.

Professor Lars Holsti contributed to the development of the field by serving in several positions of trust and as a visiting professor in Wisconsin, USA in 1974 and 1978. He was a founding member of ESTRO and belonged to the board between 1980 and 1984. As the chairman of Finnish Cancer Organizations (1973–1987) he focused on big decisions, he valued the expertise and fought against prevailing cancer pessimism. He participated in writing the Nordic plan against cancer in 1989 [3]. He received many national and international awards due to his merits in the field of cancer.

Professor Holsti laid the foundation for the high level of the current Comprehensive Cancer Center, working as the chief physician of the Department of Radiotherapy for 25 years (1968–1993). Holsti was a highly respected and liked leader with charisma, great sense of humor and strong general knowledge in addition to his professional expertise. He successfully educated the next generation of scientists and specialists in radiotherapy and oncology. His inspiring and positive spirit has been forwarded to the following generations as well.

Lasse also had his sorrows. He would have had the opportunity to tell about his experiences during war, but he remained silent, because so many did not survive.

He lost his daughter early to a serious illness, and, prematurely too, his son and his second wife Tuula (née Haggrén). But Lasse recovered by looking ahead. Lasse will be missed by his grandsons, stepson and their families and relatives. Numerous colleagues and friends have lost an excellent companion for profound discussions.
